# Molecular target based combinational therapeutic approaches in thyroid cancer

**DOI:** 10.1186/1479-5876-10-81

**Published:** 2012-05-01

**Authors:** Shilpi Rajoria, Robert Suriano, Andrea L George, Ameet Kamat, Stimson P Schantz, Jan Geliebter, Raj K Tiwari

**Affiliations:** 1 Department of Microbiology and Immunology, New York Medical College, Valhalla, New York, 10595, USA; 2 Department of Otolaryngology, New York Eye and Ear Infirmary New York, New York, 10003, USA

**Keywords:** Thyroid Cancer, Estrogen, Angiogenesis, Vascular Endothelial Growth Factor, 3,3′-diindolylmethane

## Abstract

**Background:**

Thyroid cancer, as with other types of cancer, is dependent on angiogenesis for its continued growth and development. Interestingly, estrogen has been shown to contribute to thyroid cancer aggressiveness *in vitro*, which is in full support of the observed increased incidence of thyroid cancer in women over men. Provided that estrogen has been observed to contribute to increased angiogenesis of estrogen responsive breast cancer, it is conceivable to speculate that estrogen also contributes to angiogenesis of estrogen responsive thyroid cancer.

**Methods:**

In this study, three human thyroid cancer cells (B-CPAP, CGTH-W-1, ML-1) were treated with estrogen alone or estrogen and anti-estrogens (fulvestrant and 3,3′-diindolylmethane, a natural dietary compound) for 24 hours. The cell culture media was then added to human umbilical vein endothelial cell (HUVECs) and assayed for angiogenesis associated events. Vascular endothelial growth factor (VEGF) levels were also quantified in the conditioned media so as to evaluate if it is a key player involved in these observations.

**Results:**

Conditioned medium from estrogen treated thyroid cancer cells enhanced phenotypical changes (proliferation, migration and tubulogenesis) of endothelial cells typically observed during angiogenesis. These phenotypic changes observed in HUVECs were determined to be modulated by estrogen induced secretion of VEGF by the cancer cells. Lastly, we show that VEGF secretion was inhibited by the anti-estrogens, fulvestrant and 3,3′-diindolylmethane, which resulted in diminished angiogenesis associated events in HUVECs.

**Conclusion:**

Our data establishes estrogen as being a key regulator of VEGF secretion/expression in thyroid cells which enhances the process of angiogenesis in thyroid cancer. These findings also suggest the clinical utility of anti-estrogens as anti-angiogenic compounds to be used as a therapeutic means to treat thyroid cancer. We also observed that 3,3′-diindolylmethane is a promising naturally occurring anti-estrogen which can be used as a part of therapeutic regimen to treat thyroid cancer.

## Background

The thyroid gland is a highly vascularized organ with increased vascularity observed in thyroid diseases, such as Graves’ disease, goiter and cancer, due to increased metabolic demand required for abnormal growth [1]. Common characteristics observed during this increased vascularity are enhanced endothelial cell proliferation, blood capillary enlargement, and ultimately the sprouting of new blood vessels from preexisting capillaries [2]. All of these phenotypic changes that occur are all aimed at sustaining thyroid growth and development as well as the potential of well differentiated thyroid carcinoma cells to metastasize to the regional lymph nodes, which is negatively correlated with poor prognosis and disease-free survival in patients [3,4]. This phenomenon of increased vasculature, also known as angiogenesis, is a critical and tightly regulated event that is controlled by the interplay between various cytokines and chemokines [2].

Angiogenesis involves the coordinated and directed proliferation and migration of endothelial cells, remodeling of the extracellular matrix, induction of new sprouts or tubes, anastomoses of newly formed and preexisting vessels, and basement membrane formation, in response to a stimulatory signal, such as a specific growth factor [2,5]. Cancer cells are proposed to mediate angiogenesis by secreting such growth factors that will bind to the respective receptors present on the endothelial cells resulting in endothelial cell migration, proliferation and tubulogenesis. These growth factors are termed as pro-angiogenic factors and include transforming growth factor-α (TGF-α), angiogenin, vascular permeability growth factor (VPF) and vascular endothelial growth factor (VEGF) [6]. Production of these factors causes a concomitant increase in vascularization thus leading to rapid tumor growth and metastasis. Of all the above mentioned factors involved in angiogenesis, many studies demonstrate that VEGF is a critical mediator [6-8]. VEGF is a secreted homodimeric glycoprotein that acts as a chemoattractant and mitogenic factor for endothelial cells [8,9]. There are four major members of the VEGF family, VEGF-A, -B, -C and –D with VEGF-A being the predominant form and having at least six isoforms produced through alternative splicing [10,11]. All VEGFs have differential tissue expression and bind to their tyrosine kinase cell membrane receptors, VEGF-receptor (VEGFR) -1 (also called Flt-1), VEGFR-2 (also called Flk/KDR), and VEGFR-3 (also called Flt-4) of which endothelial cells express VEGFR-1 and VEFGR-2 [12]. VEGF has been repeatedly shown as a significant component involved in regulation of thyroid angiogenesis with disturbances in intrathyroid VEGF expression leading to several thyroid pathologies, including tumor and goiter [1,13].

The expression of VEGF has been shown to be modulated by steroid hormones such as estrogens in breast cancer [14,15]. **Many*in vitro*and*in vivo*studies demonstrate that estrogen and other growth factors promote angiogenesis in endothelial cells via the estrogen receptor (ER) and that inhibition of ER regulates the intracellular balance of pro and anti-angiogenic factors**[16]**. Estrogen-ER binding also induces endothelial cell proliferation and migration opening the possibility of the use of ER competitors as possible antiangiogenic agents.** Our group has previously demonstrated an estrogen induced enhanced expression of VEGF *in vivo*, thus, participating in neo-vascularization of estrogen responsive breast tumor tissues [15]. In addition, we have recently shown that estrogen enhances thyroid cancer cell proliferation and metastasis associated events such as migration, adhesion and invasion, at least partly by induction and activation of essential proteolytic enzymes, mainly matrix metalloproteinases-2 (MMP-2) and MMP-9 [17]. The MMP system plays an important role degradation of extracellular matrix, thus not only aiding in tumor cell invasion and metastases, but also in endothelial cell migration and angiogenesis [18]. These observations along with literature suggest that estrogen is an important pro-angiogenic factor that regulates angiogenesis in hormone responsive tissues by affecting the balance of pro- and anti-angiogenic factors, but the effect of estrogen in angiogenesis of thyroid cancer is still not fully elucidated.

The rationale of this study was to determine the contribution of estrogen on angiogenesis of thyroid cancer and in order to do so, *in vitro* experiments were initiated using human thyroid cancer cells and human umbilical vein endothelial cell (HUVECs). We demonstrate that estrogen treated thyroid cancer cells secrete factors that promote phenotypic changes in HUVECs leading to enhanced migration, proliferation and tubulogenesis of endothelial cells. We also observed that these phenotypic changes in HUVECs are induced by the estrogen mediated secretion of the soluble ligand (VEGF) by cancer cells. We also investigated the effects of the anti-estrogenic compound 3,3’-diindolylmethane (DIM) on angiogenesis of thyroid cancer cells. DIM is a promising naturally available bioactive compound which can be used as an anticarcinogenic agent and anti-estrogen as it provides a safer and predictable response and has been shown to affect estrogen responsive tissues such as breast [17,19,20]. As regards to cancer prevention, several studies have demonstrated that the consumption of certain foods such as cruciferous vegetables have an inverse relationship with cancer risk. Recently, our group has discovered that DIM modulates the estrogen metabolism in thyroid proliferative disease patients, generating metabolites with anti-estrogenic activity, thus resulting in an increase in the ratio of 2-hydroxyestrones (C-2) to 16α-hydroxyestrone (C-16) [21]. We have recently demonstrated the anti-estrogenic effects of DIM on *in vitro* thyroid cancer cell proliferation, adhesion, invasion and migration [17]. These observations suggests that DIM may be a promising naturally available bioactive compound which can be used as an anticarcinogenic agent and anti-estrogen as it provides a safer and predictable response and has been shown to affect estrogen responsive tissues such as breast. In the present communication, we observe that estrogen induced angiogenesis is targeted by DIM by downregulating the bioavailability of proangiogenic factor VEGF as evidenced by reduced angiogenesis of HUVEC by DIM treated thyroid cancer cell conditioned medium. Our observations suggest that estrogen is a mediator of angiogenesis as it that might activate the formation of a paracrine loop between endothelial cells and thyroid cancer cells, which is targeted by DIM.

## Methods

### Cell culture

Three thyroid cell lines were used in this study, BCPAP (human papillary thyroid cancer cell line), CGTHW-1 (human follicular thyroid cancer cell line) and ML-1 (human follicular thyroid cancer). All thyroid cancer cells were purchased from DSMZ, Braunschweig, Germany. BCPAP and CGTHW-1 were cultured in RPMI-1640 (Mediatech, Herndon, VA) supplemented with 10% fetal bovine serum (FBS) (Atlanta Biologicals, Atlanta, GA), penicillin 10,000 IU/ml, streptomycin 10,000 μg/ml (Mediatech) and 2 mM L-glutamine (Mediatech). ML-1 was grown in DMEM (Mediatech) supplemented with 10% FBS, penicillin 10,000 IU/ml, streptomycin 10,000 μg/ml and 2 mM L-glutamine. Human Umblical Vein Endothelial Cells (HUVECs) (ATCC, Manassas, VA) were grown in FK-12 supplemented with 10% fetal bovine serum (FBS), 50 IU/ml penicillin, 50 μg/ml streptomycin, ECGS and heparin and were cultured only till passage 40.

### Conditioned medium generation

Thyroid cells were seeded at a density of 5X10^5^ cells per well in 6-well culture dishes and allowed to adhere overnight after which they were then switched to serum free medium and incubated with 10^-8^ M estrogen (E_2_) **(Sigma Chemical Company, St. Louis, MO)** ± 10^-6^ M fulvestrant **(Sigma Chemical Co.)** ± 25 μM DIM or left untreated for 24 hours. **DIM is kindly provided by Dr. Michael Zeligs (BioResponse, Boulder, Colorado) for all the experiments.** The ‘*conditioned medium*’ was then harvested and centrifuged to remove any debris and aliquots were stored at −80°C until used.

### HUVEC migration

Migration of HUVEC cells is crucial for angiogenesis and the ability of thyroid conditioned medium to modulate this event was examined using a modified Boyden chamber technique. Transwell Control Inserts with 3-μm pore membrane filters were used for HUVEC migration assay. HUVECs were harvested by trypsinization and 1x10^4^ cells were seeded in the upper chamber in 100 μl of media containing 1% FBS. The lower chamber contained 600 μl of thyroid cancer cell conditioned medium (thyroid cancer cells treated with ± 10^-8^ M E_2_ ± 10^-6^ M fulvestrant ± 25 μM DIM for 24 hours) supplemented with 1% FBS. After 6 hours of incubation, the non migrating cells were removed from the upper surface of the membrane by gently scrubbing using cotton tipped swab. Migrated cells on the lower surface of the membrane were then fixed using methanol and stained using 1% toluidine blue 1% borax stain followed by two washes with distilled water. Inserts were then allowed to airdry and counted in 10X field. Data are expressed as numbers of migrated cells per 10X field micrograph for each sample well and normalized to cell counts obtained from the untreated control.

### HUVEC proliferation assay

To evaluate the biological activity of conditioned medium obtained from thyroid cancer cells, a HUVEC proliferation assay was carried out. Fresh conditioned medium was obtained from thyroid cancer cells treated with ± 10^-8^ M E_2_ ± 10^-6^ M fulvestrant ± 25 μM DIM for 24 hours. HUVECs were harvested and seeded at a density of 1x10^4^ cells per well in 6-well culture dishes in triplicate repeats and allowed to adhere overnight. After approximately 16–18 hours, HUVECs were washed with serum free FK-12 and HUVEC growth medium was changed to various thyroid conditioned medium. After 24 hours of incubating HUVECs in thyroid cancer cell conditioned medium, HUVECs were harvested and stained using a 0.4% trypan blue solution (Sigma Chemical Co.). The number of viable (unstained) and dead (stained) cells was counted and the effect of thyroid cancer cell conditioned medium on HUVEC growth was calculated.

### HUVEC tubulogenesis

A matrigel tube formation assay, known as tubulogenesis, was performed to assess the ability of HUVECs to form endothelial cell vascular structures or tubules which is implicated in being a very crucial event for new vessel formation (angiogenesis). Individual wells were coated with 100 μl of growth factor reduced matrigel in a 96-well plate and allowed to polymerize at 37°C for 1 hour. After polymerization, 1x10^4^ HUVECs were resuspended in 100 μl thyroid cancer cells conditioned medium (thyroid cancer cells treated with ± 10^-8^ M E_2_ ± 10^-6^ M fulvestrant ± 25 μM DIM for 24 hours) were added to each well in triplicates. Cells were inspected every three hours for tubule formation and pictures were taken using light microscope at 5X.

### VEGF ELISA

The RayBio® Human VEGF ELISA (Enzyme-Linked Immunosorbent Assay) kit (RayBiotech, Inc., Norcross GA) was used for quantitative measurement of VEGF secreted in the thyroid cancer cell conditioned medium. Manufacturer’s instructions were followed for detection of VEGF in thyroid cancer cell conditioned medium. The mean absorbance for each set of standards and samples was calculated and standard curve was plotted with standards concentration on the x-axis and absorbance in the y-axis. Based on the standard curve, VEGF concentration in pg/ml was calculated.

### Neutralizing VEGF activity assay

To confirm the activity of VEGF in angiogenesis associated events of thyroid cancer cells, anti-human VEGF neutralizing antibody (R&D Systems, Inc., Minneapolis, MN) was used to neutralize VEGF bioactivity. Briefly, conditioned medium was generated by treating thyroid cancer cells for 24 hours with estrogen and/or fulvestrant as described earlier and stored at −80°C. Before the experiments, the conditioned media was incubated with 1 μg of anti-human VEGF neutralizing antibody per mL of the conditioned media at 37°C for one hour. The conditioned media with or without the VEGF neutralizing antibody was then used for tubulogenesis, proliferation and migration of HUVECs as described in the earlier section. This was to confirm that the angiogenesis associated events observed with conditioned medium of thyroid cancer cells is related to the enhanced VEGF secretion with estrogen treatments.

### Western Blot analysis

Cells were harvested using 0.25% trypsin (Mediatech), washed with PBS, and lysed (1 × 10^6^/100 μL of lysis buffer) using the radioimmunoprecipitation assay (RIPA) buffer [50 mM Tris–HCl (pH 7.4), 150 mM NaCl, 0.2% sodium deoxycholate, 0.1% SDS, 0.5% NP40, 1 μM Pefabloc] and incubated on ice for 30 minutes with intermittent vortexing. The lysates were centrifuged at 14,000 rpm for 30 min at 4°C and supernatant were collected. Cell lysates (20 μg protein) were subjected to 12% SDS–PAGE under reducing conditions (presence of β-mercaptoethanol) as described earlier [17]. Briefly, the proteins were transferred to Immobilon-P membranes at 220 mA for 2 h and membranes were blocked with 4% dried milk in TBST [200 mM Tris–HCl, pH 7.4, 150 mM NaCl, and 0.01% Tween-20 added fresh/liter of 1×TBS (TBST)] for at least 2–3 h on a shaker at room temperature. Subsequently, the membranes were incubated overnight at 4°C with either VEGF (Santa Cruz Biotechnology, Santa Cruz, CA) antibody or actin (Santa Cruz Biotechnology) in TBST. Membranes were washed three times with TBST and incubated with the respective horseradish peroxidase (HRP) conjugated secondary antibody, for 2 hours at room temperature in TBST containing 2% milk. After four washes with TBS-T and one wash with TBS, membranes were developed by ECL substrate (Pierce Rockford, IL) and detected on Denville autoradiography films.

### Statistical Calculation

Experiments presented here represent means of two to four replicates with statistical significance determined using a paired Student’s *t* test and one-way ANOVA followed by Tukey’s multiple comparison tests. The probability (‘*p*’ value) ≤ 0.05 was used to reject the null hypothesis in all the experiments.

## Results

### Estrogen treated thyroid cancer cells enhance migration of endothelial cells

**Figure 1:**
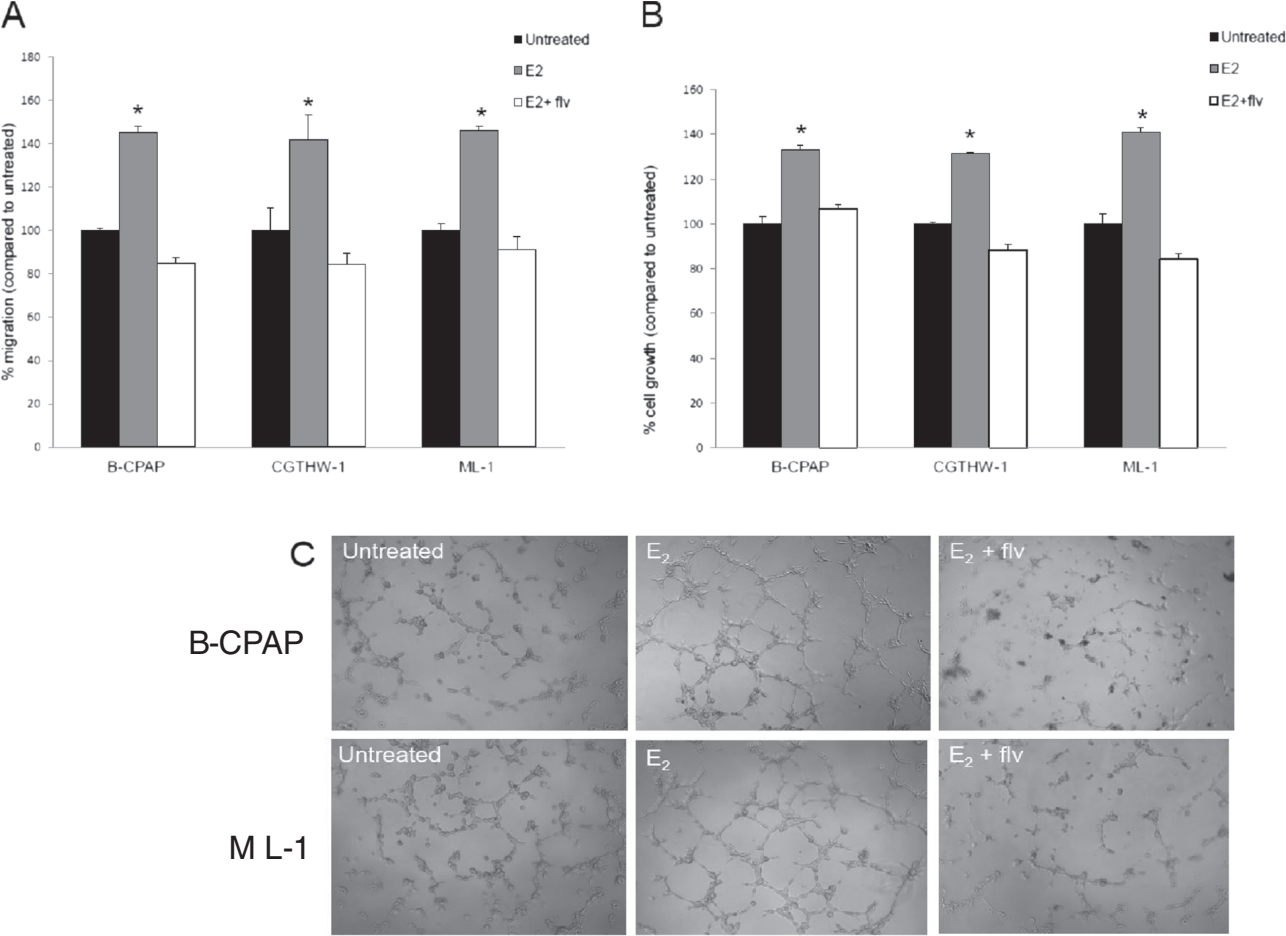
**Estrogen treated thyroid cancer cells enhance angiogenesis of HUVECs.** (**A**) Estrogen treated thyroid cancer cells enhance HUVEC migration. Conditioned medium from thyroid cancer cells with 10^-8^ M E_2_ ± 10^-6^ M fulvestrant was used as chemoattractant for HUVECs. The HUVECs that migrated and adhered on the lower surface of the membrane were fixed, stained, and counted in 10X field. The groups are as follows- HUVECs migrated to the untreated (black bars), E_2_ treated (grey bars) and E_2_ and fulvestrant treated (white bars) conditioned medium. Data expressed as numbers of HUVECs counted (migrated cells) per 10X field micrograph for each sample well and normalized to the untreated control set as 100%. The asterisk denotes statistically significant differences (*p* < 0.05) between experimental and untreated samples determined using a paired Student’s *t -* test. (**B**) Estrogen treated thyroid cancer cells enhance HUVEC proliferation. HUVECs were cultured in presence of thyroid cancer cell conditioned medium for 24 hours followed by trypan blue exclusion cell count to calculate endothelial cell proliferation. The groups are as follows- HUVECs cultured with untreated (black bars), E_2_ treated (grey bars) and E_2_ and fulvestrant treated (white bars) conditioned medium. Data expressed as % of HUVECs cell number normalized to the untreated controls set as 100%. The asterisk denotes statistically significant differences (*p* < 0.05) between experimental and untreated samples determined using a paired Student’s *t –* test. (**C**) Enhances tube formation of HUVECs induced by estrogen treated thyroid cancer cell conditioned medium. HUVECs were seeded in B-CPAP and ML-1 thyroid cancer cell conditioned medium on Growth factor-reduced Matrigel and were monitored for tubulogenesis. The results are representative of three independent experiments performed in triplicates.

In order to investigate the pro-angiogenic effects of estrogen in TPD, we first evaluated HUVEC migratory function using a modified Boyden chamber technique. HUVECs were cultured in transwell inserts with conditioned media from thyroid cancer cells treated with estrogen ± fulvestrant in the lower chamber. We observed that the migration of HUVECs to estrogen treated thyroid cancer cell conditioned medium was significantly higher than that of untreated controls (Figure 1A). This increase with estrogen treatment was approximately 45% for B-CPAP conditioned medium, 41% for CGTH-W-1 conditioned medium and 46% for ML-1 thyroid cancer cell conditioned medium. More interestingly, this estrogen enhanced increase in migration of HUVECs was abrogated in the presence of fulvestrant. This decrease in migration with fulvestrant and estrogen treated thyroid cancer cell conditioned medium was 8-16% of untreated cells and was cell line dependent. The fact that thyroid cancer cell conditioned medium, even untreated thyroid cancer cell conditioned medium, contains chemoattractants for endothelial cells has interesting implications with respect to thyroid cancer and how it is capable of initiating the event of its own blood supply as well as establishing secondary foci during metastasis.

### Conditioned medium from estrogen treated thyroid cancer cells enhance proliferation of endothelial cells

To examine the growth stimulation of HUVECs in response to thyroid cancer cell conditioned medium, we cultured HUVECs in presence of conditioned medium collected from thyroid cancer cells for 24 hours. The number of HUVECs in culture with thyroid cancer cell conditioned medium from cells treated with estrogen was significantly higher than that of the untreated thyroid cancer cell conditioned media (controls) (Figure 1B). The increase in proliferation with estrogen treated thyroid cancer cell conditioned medium was 33% for B-CPAP, 31% for CGTH-W-1 and 41% for ML-1. This estrogen enhanced cell proliferation was down-regulated when thyroid cancer cells were treated with fulvestrant along with estrogen. These experiments suggest that proliferation of HUVECs is associated with mitogenic soluble factor(s) secreted by thyroid cancer cells when treated with estrogen.

### Enhanced tubulogenesis of HUVECs is induced by conditioned media from estrogen treated thyroid cancer cells

To investigate the pro-angiogenic effects of estrogen in TPD with respect to this tubular network formation, an *in vitro* tubulogenesis assay was performed using HUVECs (Figure 1C). HUVECs were cultured on the Matrigel coated plates in the presence of conditioned medium collected from thyroid cancer cells treated with estrogen ± fulvestrant for the period of four hours. Matrigel^TM^ is a basement membrane matrix containing collagen, fibronectin and laminin and it mimics an *in vivo* basement membrane [22]. Culturing HUVECs on this substrate allowed us to observe how the endothelial cells behave in response to the tumor cells secreted factors. We discovered that conditioned medium from B-CPAP and ML-1 thyroid cancer cells treated with estrogen can stimulate the formation of well defined capillary-like structures by HUVECs on matrigel, while HUVECs formed short stumped structures on matrigel when cultured with estrogen and fulvestrant treated thyroid cancer cell conditioned media. This enhanced tubulogenesis of HUVECs with estrogen treated thyroid cancer cell conditioned media was not due to proliferation of HUVECs as the number of HUVECs was not affected in six hours.

### Estrogen induces VEGF secretion of thyroid cancer cells

**Figure 2:**
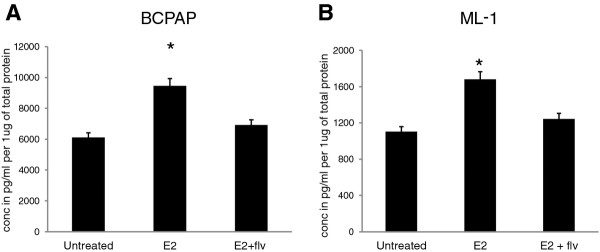
**Effect of estrogen on the* in vitro *VEGF secretion by thyroid cancer cells.** B-CPAP and ML-1 thyroid cancer cells were seeded at a density of 5X10^5^ cells per well in six-well culture dishes and allowed to adhere overnight. Thyroid cancer cells were then switched to serum free medium and incubated with ± 10^-8^ M E_2_ ± 10^-6^ M fulvestrant or left untreated for 24 hours. The conditioned medium was harvested and (**A**) B-CPAP and (**B**) ML-1 conditioned medium was assayed for VEGF secretion using VEGF ELISA kit. The asterisk denotes statistically significant differences (*p* < 0.05) between experimental and untreated samples determined using a paired Student’s *t -* test. The standard deviation represents n = 3 for each sample.

To investigate whether VEGF acts as an effector molecule for estrogen stimulated angiogenic phenotypic characteristic of HUVECs, we quantified *in vitro* VEGF secretion by B-CPAP (Figure 2A) and ML-1 (Figure 2B) in response to estrogen and fulvestrant by a quantitative VEGF-ELISA. Basal *in vitro* secretion of VEGF (untreated) was 6108 pg/ml in B-CPAP and 1103 pg/ml in ML-1. When treated with estrogen, the thyroid cancer cells displayed an enhanced VEGF secretion with 9455 pg/ml in B-CPAP and 1681 pg/ml in ML-1, which corresponds to a significant increase (50-55%) in VEGF secretion upon estrogen treatment. With fulvestrant, the VEGF secretion was reverted back to untreated levels. These enhanced levels of VEGF potentially increase the endothelial cells ability to establish neovasculature, as evidenced by the observed *in vitro* migration, proliferation and tubulogenesis of endothelial cells.

### Blocking secreted VEGF suppresses angiogenesis associated events in thyroid cancer cells

**Figure 3:**
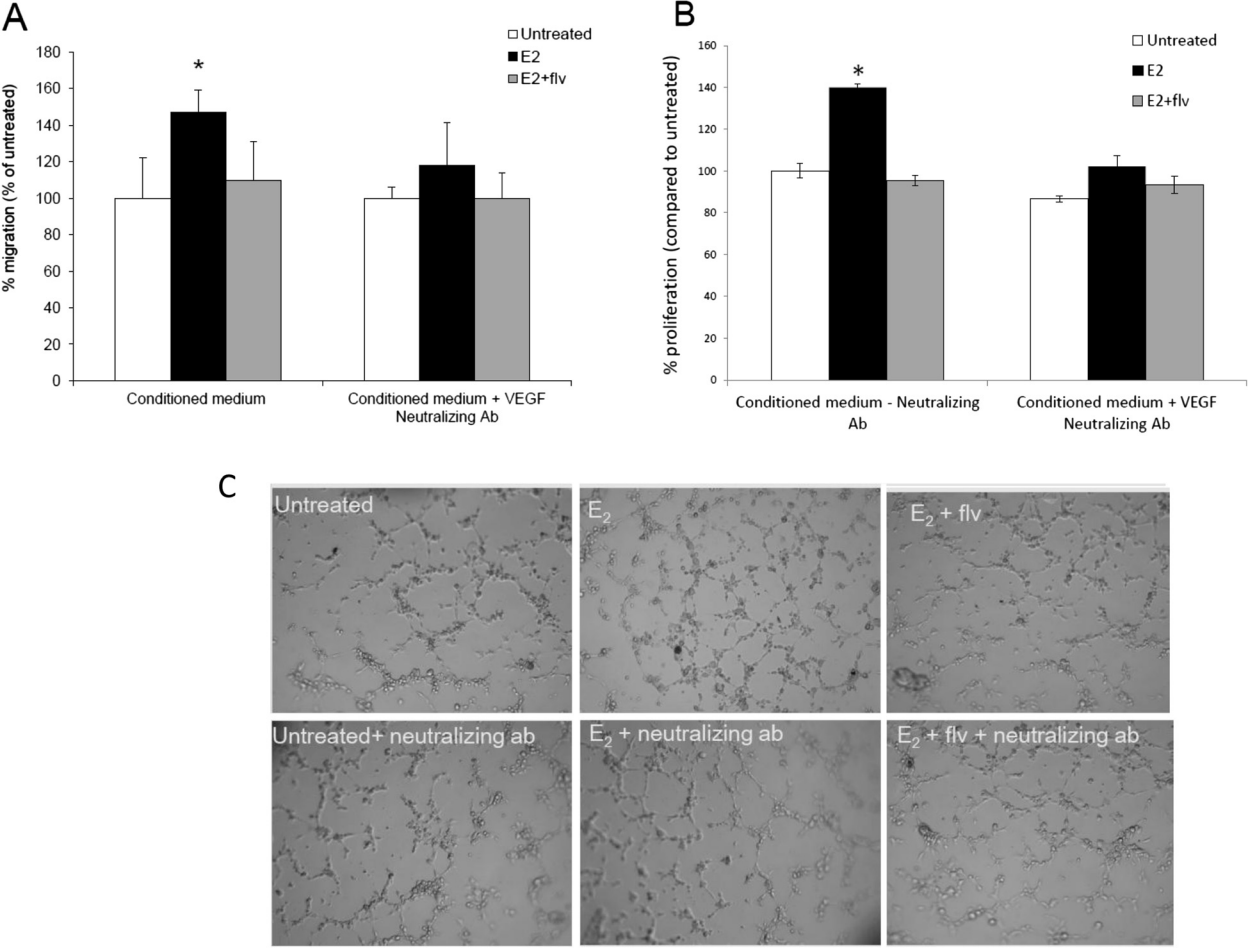
**HUVEC migration, proliferation and tubulogenesis are suppressed by VEGF neutralizing antibody.** Conditioned medium generated by culturing ML-1 thyroid cancer cells with ± 10^-8^ M E_2_ ± 10^-6^ M fulvestrant was incubated with 1 μg of anti-human VEGF neutralizing antibody per mL of the conditioned media at 37°C for one hour. (**A**) HUVECs were allowed to migrate towards thyroid cancer cell conditioned medium with or without the VEGF neutralizing antibody used as chemoattractant. Data expressed as numbers of HUVECs counted (migrated cells) per 10X field micrograph for each sample well and normalized to the untreated control. The asterisk denotes statistically significant differences (*p* < 0.05) between experimental and untreated samples determined using a paired Student’s *t -* test. (**B**) HUVECs were cultured in presence of thyroid cancer cell conditioned medium followed by trypan blue exclusion cell count to calculate endothelial cell proliferation. Data expressed as % of HUVECs cell number normalized to the untreated control **(no neutralizing antibody)** set as 100%. The asterisk denotes statistically significant differences (*p* < 0.05) between experimental and untreated samples determined using a paired Student’s *t -* test. (**C**) HUVECs were seeded in ML-1 thyroid cancer cell conditioned medium with or without the VEGF neutralizing antibody on matrigel and monitored for tubulogenesis. The results are representative of three independent experiments performed in triplicates.

 To further validate the role of VEGF in enhanced angiogenesis of thyroid cancer cells and understand the mechanism by which estrogen regulates angiogenesis, we neutralized secreted VEGF in the conditioned medium by using an anti-human neutralizing VEGF antibody. Free secreted VEGF can bind to the VEGF-receptor (VEGFR) present on endothelial cells enabling them to migrate, proliferate and form tubules. By using a neutralizing VEGF antibody, we blocked this step of angiogenesis. Thyroid cancer cells were incubated with estrogen and fulvestrant to generate conditioned medium, which was evaluated for VEGF secretion. One microgram of neutralizing VEGF antibody was then incubated with the conditioned medium to neutralize free-VEGF. This conditioned medium was then subjected to HUVEC migration, proliferation and tubulogenesis to confirm the effects of VEGF on thyroid cancer angiogenesis. Conditioned medium, without neutralizing antibody, was also used as an internal control. We discovered that in presence of neutralizing antibody, the migration of HUVECs in response to estrogen treated thyroid cancer cell conditioned medium was suppressed (Figure 3A). We observed a significant increase (45-55%) in migration of HUVECs with estrogen treated ML-1 conditioned medium, which was suppressed to only 16-18% when secreted VEGF was neutralized. The HUVEC proliferation in response to estrogen treated thyroid cancer cell conditioned medium was also down-regulated when secreted VEGF was neutralized (Figure 3B). Also, a similar suppression in HUVEC tubulogenesis was observed with VEGF neutralizing antibody (Figure 3C). This further clarified that the induction in angiogenesis associated events by estrogen is due to enhanced secretion of VEGF in combination with other growth factors not currently identified in this study, which may explain only a partial inhibition in the angiogenesis associated events in the presence of the neutralizing VEGF antibody.

### Dietary compound DIM inhibits estrogen induced angiogenesis of thyroid cancer cells

**Figure 4:**
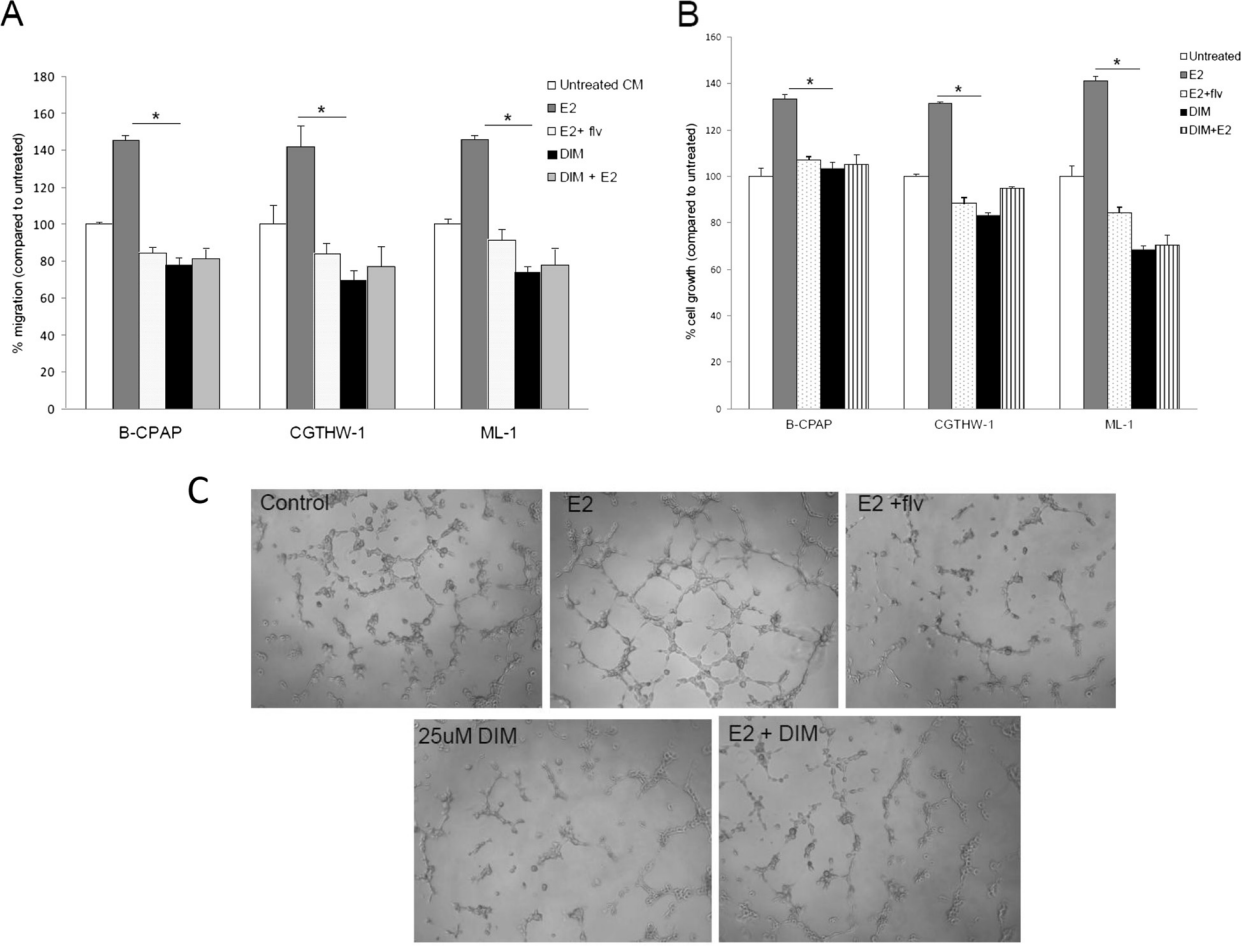
**Estrogen enhanced HUVEC migration, proliferation and tubulogenesis are attenuated by DIM.** (**A**) Conditioned medium was generated by culturing thyroid cancer cells with 10^-8^ M E_2_ ± 25 μM DIM or left untreated for 24 hours. HUVECs were allowed to migrate towards thyroid cancer cell conditioned medium used as chemoattractant. The HUVECs that migrated and adhered on the lower surface of the membrane were fixed, stained, and counted in 10X field. The groups are as follows- HUVECs migrated to the untreated (white bars), E_2_ treated (grey bars), E_2_ and fulvestrant treated (dotted bars), 25 μM DIM (black bars) and 25 μM DIM + E_2_ conditioned medium (striped bars). Data expressed as numbers of HUVECs counted (migrated cells) per 10X field micrograph for each sample well and normalized to the untreated control. (**B**) HUVECs were cultured in presence of thyroid cancer cell conditioned medium followed by trypan blue exclusion cell count to calculate endothelial cell proliferation. The groups are as follows- HUVECs migrated to the untreated (white bars), E_2_ treated (grey bars), E_2_ and fulvestrant treated (dotted bars), 25 μM DIM (black bars) and 25 μM DIM + E_2_ conditioned medium (striped bars). Data expressed as % of HUVECs cell number normalized to the untreated control. The asterisk denotes statistically significant differences (*p* < 0.05) between experimental and untreated group using one way ANOVA tests. (**C**) Conditioned medium was generated by culturing ML-1 cells with estrogen ± DIM for 24 hours. Growth factor-reduced Matrigel was plated and allowed to polymerize in 96-well plates. HUVECs were seeded in ML-1 thyroid cancer cell conditioned medium on matrigel. The plate was incubated for four to six hours and the wells were photographed using an inverted microscope with a digital camera. The results are representative of three independent experiments performed in triplicates.

To determine whether conditioned medium from DIM treated thyroid cancer cells could reduce angiogenesis associated events in HUVECs, we performed *in vitro* angiogenesis assay of HUVECs. Firstly, as shown in Figure 4A and additional file 1, HUVECs migration is reduced towards DIM treated samples compared to untreated samples. More interestingly, DIM was able to down-regulate the estrogen induced HUVEC migration (compare grey and striped bars). Moreover, HUVEC proliferation was suppressed in the presence of conditioned medium from DIM treated thyroid cancer cells. Especially for ML-1, proliferation of HUVECs was significantly down-regulated for conditioned medium from DIM and DIM + estrogen treated thyroid cancer cells (Figure 4B and additional file 1). Furthermore, tubulogenesis of endothelial cells (HUVECs) was performed with conditioned medium generated from thyroid cells treated with DIM ± estrogen. We discovered that HUVECs formed irregular structures on matrigel when cultured with DIM-conditioned media with no well defined tubules even when DIM and estrogen treatments were combined (Figure 4C). The effect of DIM on estrogen mediated tubulogenesis was comparable to activity of fulvestrant, suggesting that DIM possess not only anti-estrogenic activity but also anti-angiogenic activity as well.

### DIM down-regulates estrogen enhanced VEGF secretion

**Figure 5:**
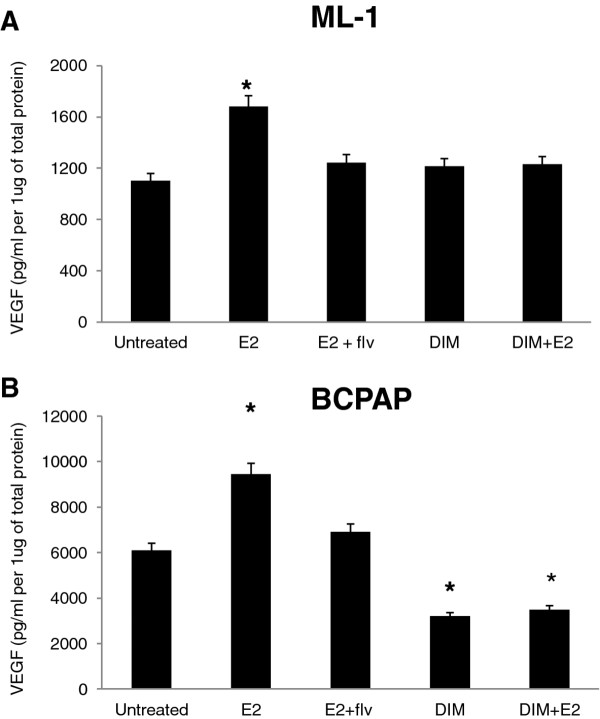
**DIM targets VEGF secretion of thyroid cancer cells. B-CPAP and ML-1 thyroid cancer cells were seeded at a density of 5×10**^**5**^** cells per well in six-well culture dishes and allowed to adhere overnight.** Thyroid cancer cells were then switched to serum free medium and incubated with ± 10^-8^ M E_2_ ± 10^-6^ M fulvestrant or left untreated for 24 hours. The conditioned medium was harvested and (**A**) ML-1 and (**B**) B-CPAP VEGF was assayed using VEGF ELISA kit. The asterisk denotes statistically significant differences (*p* < 0.05) between the treatment and untreated samples determined using a one way ANOVA test. The standard deviation represents n = 3 for each sample.

We have recently shown that DIM targets estrogen enhanced MMP secretion [17] and estrogen enhances *in vitro* angiogenesis associated events (current study), thus to examine the mechanism of action of DIM as an inhibitor of estrogen enhanced angiogenesis, we determined whether DIM inhibited VEGF secretion of thyroid cancer cells. Conditioned medium from thyroid cancer cells treated with DIM ± estrogen was quantified for *in vitro* VEGF secretion of B-CPAP and ML-1 (Figure 5A, 5B and additional file 2) in response to estrogen and DIM by VEGF-ELISA. We discovered that the levels of VEGF were down-regulated for DIM treated samples, suggesting that the decreased levels of VEGF in conditioned medium by DIM treatments may be (at least in part) responsible for reduced angiogenesis of HUVECs.

### DIM modulates VEGF expression in HUVECs

**Figure 6:**
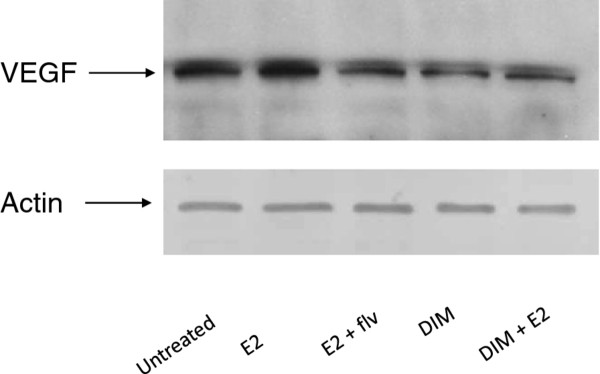
**DIM down-regulates estrogen enhanced VEGF expression of HUVECs. ML-1 thyroid cancer cells were seeded at a density of 5×10**^**5**^** cells per well in six-well culture dishes and allowed to adhere overnight.** Thyroid cancer cells were then switched to serum free medium and incubated with ± E_2_ ± DIM or left untreated for 24 hours. The conditioned medium was harvested and HUVECs were cultured in presence of this conditioned medium for 24 hours, followed by whole cell lysate formation. Subsequently, Western blot analysis was performed using anti-VEGF antibody to analyze VEGF expression in HUVECs.

To further explore the ability of DIM to disrupt angiogenesis, we treated HUVECs with thyroid cancer cell conditioned medium for 24 hours, followed by Western blot analysis of whole HUVEC cell lysates (Figure 6). We found that DIM successfully inhibits the expression of VEGF in HUVECs when thyroid cells were treated with DIM, which may be due to reduced levels of VEGF and other growth factors released by thyroid cancer cells in conditioned medium when treated with DIM. These reduced levels of mitogenic factors potentially weaken the ability of endothelial cells to establish vascularization. Our data suggests a strong link between estrogen and angiogenesis as evidenced by *in vitro* models and the ability of a natural non-toxic dietary agent, DIM, to inhibit any direct effect of estrogen on thyroid cells thus allowing the possible development of an anti-estrogenic therapy for thyroid malignancies.

## Discussion

Certain conditions such as inflammation, hypoxia and genetic lesions are known to modulate the angiogenic switch which comprises of a balance between positive and negative regulators [23-25]. We have earlier demonstrated that estrogen is a promoter of thyroid cancer. In this study, we provided evidences that the higher incidence of thyroid cancer in females is potentially attributed to the presence of a functional ER that participates in cellular processes contributing to enhanced mitogenic and metastatic properties of thyroid cells [26]. We have also demonstrated that DIM targets estradiol induced thyroid cancer cell proliferation and metastasis associated events, namely adhesion, migration and invasion, potentially by targeting the metastatic modulators MMP-2/MMP-9 [17], that not only regulate angiogenesis but are also required for degradation of collagen, a major component of extracellular matrices [27,28]. MMPs have also shown to enhance the bioavailability of VEGF by proteolytically cleaving the extracellular matrix bound VEGF allowing the association between VEGF and its respective receptor, VEGFR [28-31]. This association between growth factor and its respective receptor aids in the generation of tumor microenvironment which is conducive to tumor growth and angiogenesis [28,30,31]. These observations suggest the potential role of estrogen in mediating angiogenesis of thyroid cancer cells.

We provide evidence in this report that estrogen treatment leads to enhanced levels of VEGF secretion by thyroid cancer cells, thus leading to enhanced angiogenesis associated events, including migration, proliferation and tubulogenesis of endothelial cells cultured in presence of thyroid cancer cell conditioned medium. We discovered that thyroid cancer cells and endothelial cells *crosstalk* via growth factors such as VEGF. Many studies demonstrate that VEGF plays a central and critical role in angiogenesis, regulating the expression of angiogenesis related genes in endothelial cells and the proliferation, migration as well as tubulogenesis of endothelial cells [6,7,9,12,15]. These studies demonstrating the contribution of VEGF were validated in the presence of VEGF neutralizing antibody.

We further evaluated the effects of DIM on angiogenesis of thyroid cancer. Therapeutic and preventive strategies that target VEGF such as anti-VEGF antibodies and soluble VEGF receptors have been shown to reduce growth of several cancers but thyroid. We present in the current report that the estrogen induced angiogenesis is targeted by anti-estrogen DIM by downregulating the bioavailability of proangiogenic factor VEGF as evidenced by reduced angiogenesis of HUVEC by DIM treated thyroid cancer cell conditioned medium. The ability of an anti-estrogen to inhibit any direct effect of estrogen on thyroid cancer cells suggests that the estrogen receptor is a viable target for treatment of thyroid cancer by way of using anti-estrogens in therapeutic and preventive settings. This was potentially due to reduced levels of secreted VEGF with anti-estrogen treatments. These reduced levels of mitogenic factors such as VEGF, potentially weaken the ability of endothelial cells to establish structures essential to vascularization of tissues, suggesting the usefulness of anti-estrogens such as DIM for treatment against aggressive thyroid carcinomas. The anti-angiogenic effects of DIM on estrogen induced migration, proliferation and tubulogenesis of endothelial cells, which leads to reduced angiogenesis shows a strong potential for use of DIM in thyroid cancer treatment and has been identified as a potential antitumor therapies candidate. Further *in vivo* experiments for the usefulness of DIM in controlling the angiogenesis of thyroid tumor are underway by our group.

## Conclusion

In its entirety, the results from the current study suggest a strong link between estrogen and angiogenesis of thyroid cancer as evidenced by *in vitro* observations. Considering that thyroid vascularization is seminal for pathogenesis of thyroid proliferative disease, our findings may have clinical utility in targeting endothelial cells for antitumor therapies, thus disrupting tumor vasculogenesis and aggressive thyroid tumors. Our results demonstrate the primary role of estradiol in modulating discrete events of angiogenesis in thyroid cells and that all of these cells have the propensity to be estrogen modulated. Our cell culture model using thyroid epithelial cancer cells and endothelial cells establish a cell-cell interactive system that highlights the role of estrogen in cancer progression. It delineates an important epidemiological observation at the cellular tumor microenvironment level. Combinational therapies that employ anti-angiogenic and anti-estrogenic agents, such as DIM and anti-VEGF, used in this study, are viable treatment and preventive strategies and are a model for the translation of *in vitro* molecular target based identification that can affect clinical practice.

## Abbreviations

VEGF: Vascular endothelial growth factor; DIM: 3,3′-diindolylmethane; TGF-α: Transforming growth factor-α; VPF: Vascular permeability growth factor; VEGFR: VEGF-receptor; MMP: Matrix metalloproteinases; HUVECs: Human umbilical vein endothelial cell; FBS: Fetal bovine serum; E2: Estrogen; ELISA: Enzyme-Linked Immunosorbent Assay; TMB: 3,3’,5,5’-tetramethylbenzidine; RIPA: Radioimmunoprecipitation assay; HRP: Horseradish peroxidase; ER: Estrogen receptor.

## Competing interests

The authors declare that they have no competing interests.

## Authors contributions

SR, RS, AG, AK, RKT conceived and designed the experiments. SR, AG, AK performed the experiments. SR, RS, SS, JG, RKT analyzed the data. SS, JG, RKT contributed reagents/material/analysis tools. SR, RS, RKT wrote the manuscript. All authors read and approved the final manuscript.

## Supplementary Material

Additional file 1 **Figure S1** Fulvestrant does not affect HUVEC migration and proliferation. (**A**) Conditioned medium was generated by culturing thyroid cancer cells with 10^-8^ M E_2_ ± 25 μM DIM ± 10^-6^ M Fulvestrant or left untreated for 24 hours. HUVECs were allowed to migrate towards thyroid cancer cell conditioned medium used as chemoattractant. The HUVECs that migrated and adhered on the lower surface of the membrane were fixed, stained, and counted in 10X field. The groups are as follows- HUVECs migrated to the untreated (white bars), E_2_ treated (grey bars), E_2_ and fulvestrant treated (dotted bars), 25 μM DIM (black bars), 25 μM DIM + E_2_ conditioned medium (striped bars) and fulvestrant treated (light gray bars). Data expressed as numbers of HUVECs counted (migrated cells) per 10X field micrograph for each sample well and normalized to the untreated control. (**B**) HUVECs were cultured in presence of thyroid cancer cell conditioned medium followed by trypan blue exclusion cell count to calculate endothelial cell proliferation. The groups are as follows- HUVECs migrated to the untreated (white bars), E_2_ treated (grey bars), E_2_ and fulvestrant treated (dotted bars), 25 μM DIM (black bars), 25 μM DIM + E_2_ conditioned medium (striped bars) and fulvestrant treated (light gray bars). Data expressed as % of HUVECs cell number normalized to the untreated control. The asterisk denotes statistically significant differences (*p* < 0.05) between experimental and untreated group using one way ANOVA tests Click here for file

Additional file 2 **Figure S2** Fulvestrant does not affect VEGF secretion of thyroid cancer cells. B-CPAP and ML-1 thyroid cancer cells were seeded at a density of 5X10^5^ cells per well in six-well culture dishes and allowed to adhere overnight. Thyroid cancer cells were then switched to serum free medium and incubated with ± 10^-8^ M E_2_ ± 10^-6^ M fulvestrant or left untreated for 24 hours. The conditioned medium was harvested and (**A**) ML-1 and (**B**) B-CPAP VEGF was assayed using VEGF ELISA kit. The asterisk denotes statistically significant differences (*p* < 0.05) between the treatment and untreated samples determined using a one way ANOVA test. The standard deviation represents n = 3 for each sample Click here for file
