# Supplementary figures and images for: Molecular target based combinational therapeutic approaches in thyroid cancer
Source: J Transl Med. 2012 May 1;10:81. doi: 10.1186/1479-5876-10-81 (PMC3418191; doi:10.1186/1479-5876-10-81)

Supplementary  
Fig 1

**A**

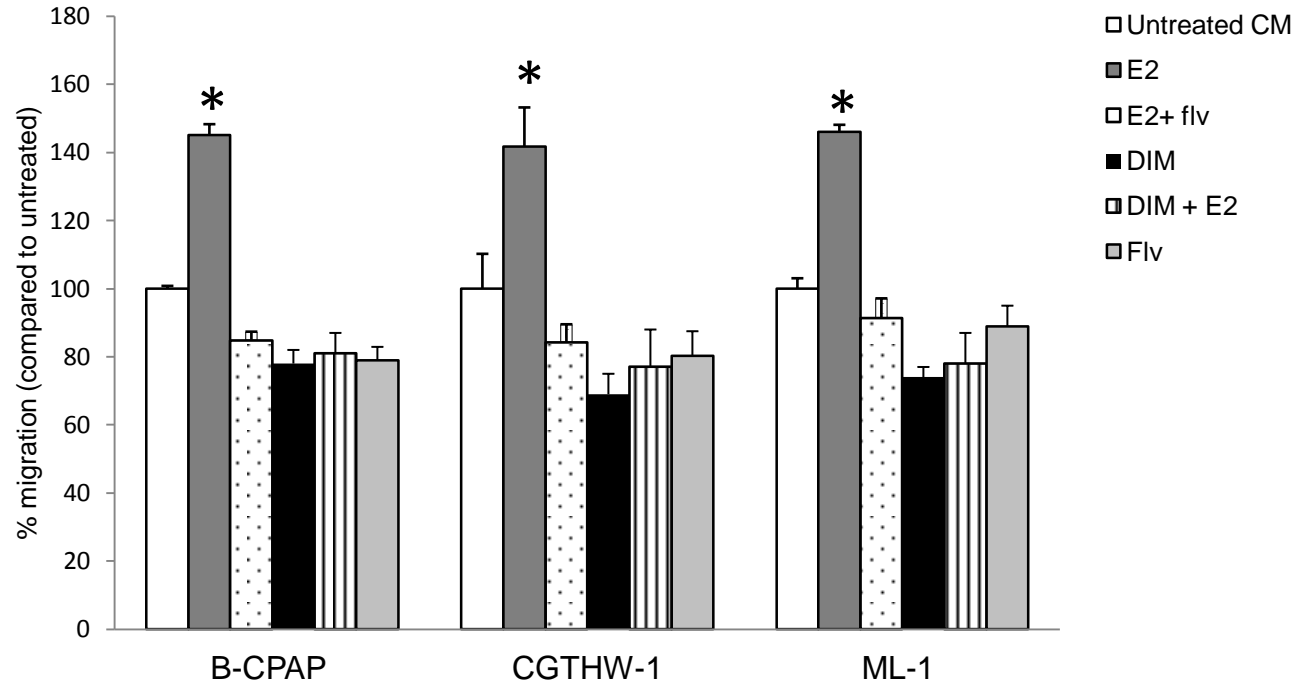

**B**

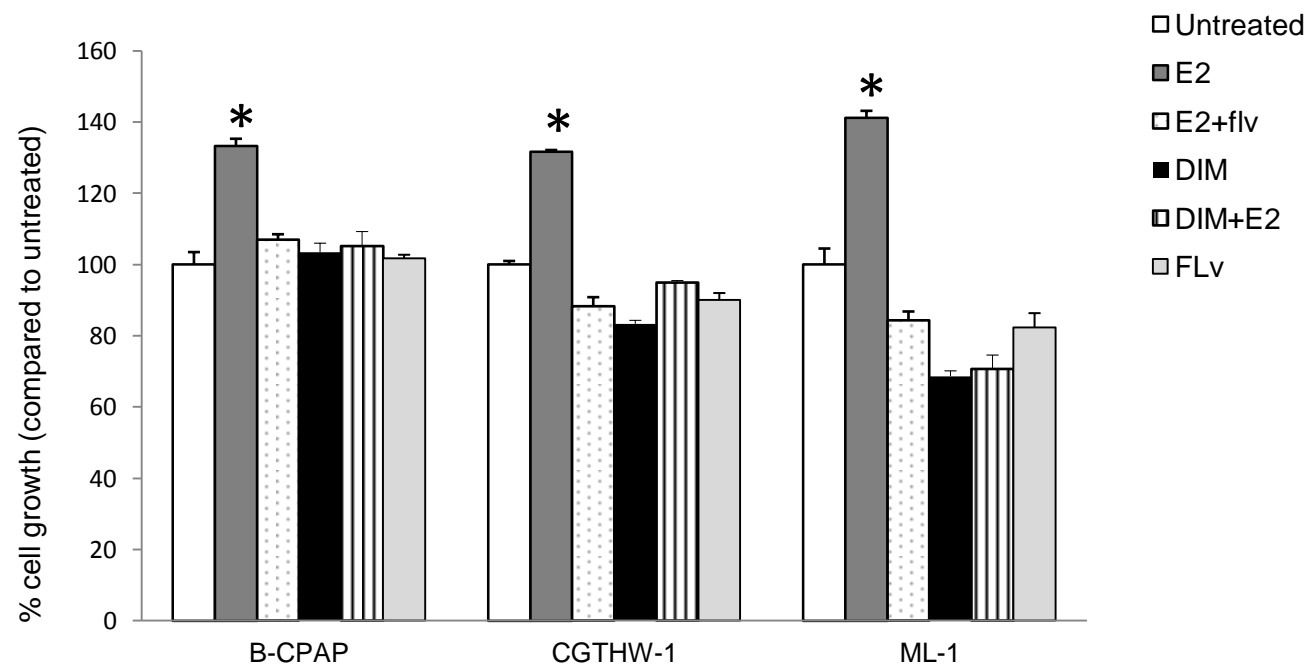

Supplement: Additional file 1 — Figure S1 Fulvestrant does not affect HUVEC migration and proliferation. (A) Conditioned medium was generated by culturing thyroid cancer cells with 10-8 M E2 ± 25 μM DIM ± 10-6 M Fulvestrant or left untreated for 24 hours. HUVECs were allowed to migrate towards thyroid cancer cell conditioned medium used as chemoattractant. The HUVECs that migrated and adhered on the lower surface of the membrane were fixed, stained, and counted in 10X field. The groups are as follows- HUVECs migrated to the untreated (white bars), E2 treated (grey bars), E2 and fulvestrant treated (dotted bars), 25 μM DIM (black bars), 25 μM DIM + E2 conditioned medium (striped bars) and fulvestrant treated (light gray bars). Data expressed as numbers of HUVECs counted (migrated cells) per 10X field micrograph for each sample well and normalized to the untreated control. (B) HUVECs were cultured in presence of thyroid cancer cell conditioned medium followed by trypan blue exclusion cell count to calculate endothelial cell proliferation. The groups are as follows- HUVECs migrated to the untreated (white bars), E2 treated (grey bars), E2 and fulvestrant treated (dotted bars), 25 μM DIM (black bars), 25 μM DIM + E2 conditioned medium (striped bars) and fulvestrant treated (light gray bars). Data expressed as % of HUVECs cell number normalized to the untreated control. The asterisk denotes statistically significant differences (p < 0.05) between experimental and untreated group using one way ANOVA tests [file 1479-5876-10-81-S1.pdf]

Fig 2

**A**

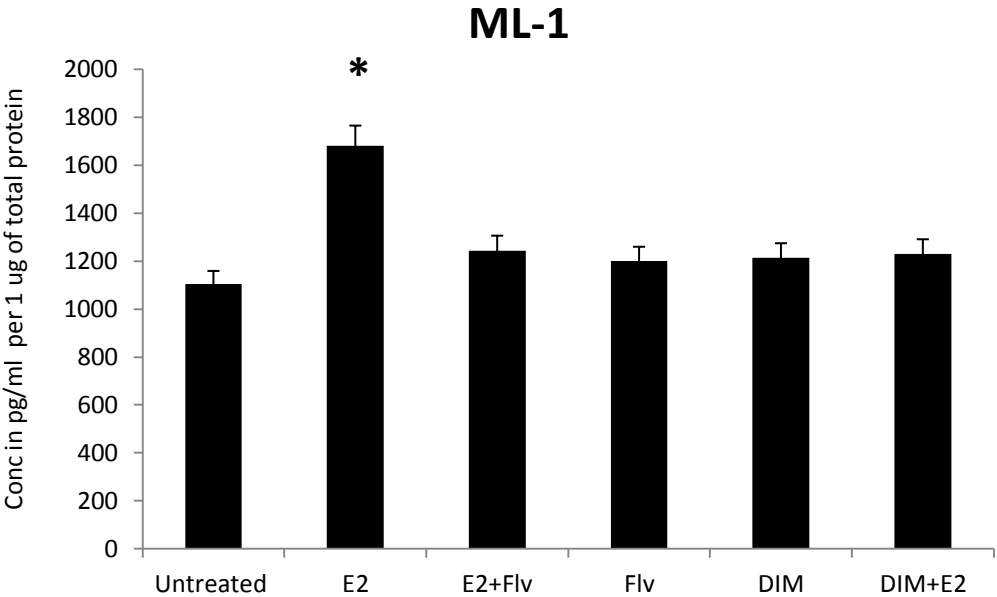

**B**

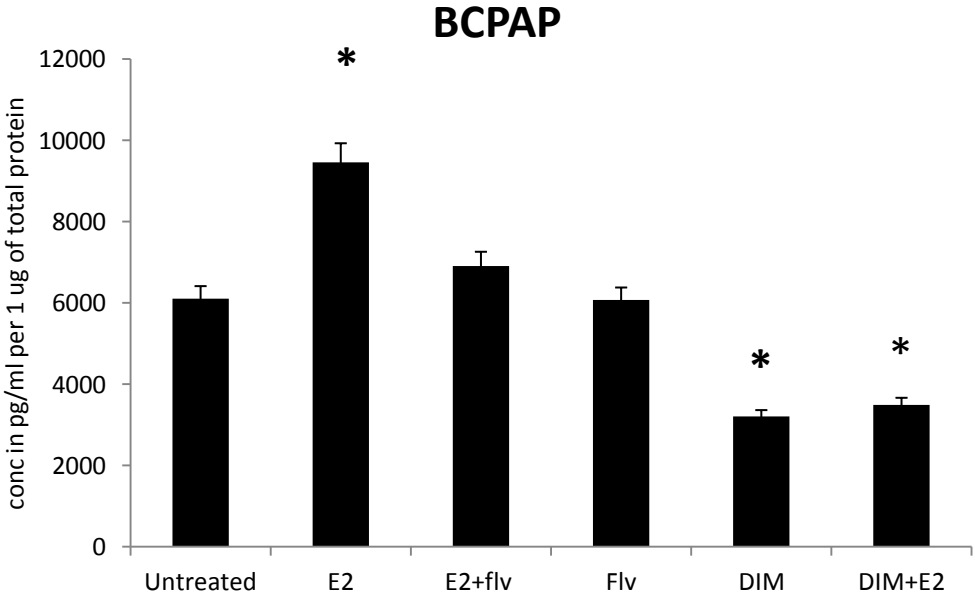

Supplement: Additional file 2 — Figure S2 Fulvestrant does not affect VEGF secretion of thyroid cancer cells. B-CPAP and ML-1 thyroid cancer cells were seeded at a density of 5X105 cells per well in six-well culture dishes and allowed to adhere overnight. Thyroid cancer cells were then switched to serum free medium and incubated with ± 10-8 M E2 ± 10-6 M fulvestrant or left untreated for 24 hours. The conditioned medium was harvested and (A) ML-1 and (B) B-CPAP VEGF was assayed using VEGF ELISA kit. The asterisk denotes statistically significant differences (p < 0.05) between the treatment and untreated samples determined using a one way ANOVA test. The standard deviation represents n = 3 for each sample [file 1479-5876-10-81-S2.pdf]
